# Sociodemographic and clinical characteristics of early COVID-19 deaths in Almadinah Almonawarah, Saudi Arabia: An analytical cross-sectional study

**DOI:** 10.12669/pjms.39.3.6736

**Published:** 2023

**Authors:** Amal M. Qasem Surrati, Eman Sobh, Farah Asad Mansuri, Abdulraouf A. Bokhari, Samira Mousa Haroon, Nouf Moalla Alewi

**Affiliations:** 1 Amal M. Qaseem Surrati, Family and Community Medicine, College of Medicine, Taibah University, Medina, Saudi Arabia; 2 Eman Sobh, Respiratory Therapy Dept., College of Medical Rehabilitation Sciences, Taibah University, Medina, Saudi Arabia. Chest Diseases Department, Faculty of Medicine for Girls, Al-Azhar University, Cairo, Egypt; 3 Farah Asad Mansuri, Family and Community Medicine, College of Medicine, Taibah University, Medina, Saudi Arabia; 4 Abdulraouf A. Bokhari, King Salaman Bin Abdulaziz Medical City, Medina, Saudi Arabia; 5 Samira Mousa Haroon, Ohud General Hospital, MOH, Medina, Saudi Arabia; 6 Nouf Moalla Alewi, Ohud General Hospital, MOH, Medina, Saudi Arabia

**Keywords:** COVID-19 early deaths, SARS-COV-2, Coronavirus, Mortality, Comorbidities, Saudi Arabia

## Abstract

**Background and Objective:**

Identification of clinical characteristics and risk factors for mortality in COVID-19 is important for early detection and precise case management. The study aimed to describe the sociodemographic, clinical, and laboratory characteristics of in-hospital COVID-19 deaths in Almadinah Almonawarah city, Saudi Arabia, and to identify risk factors for early mortality among them.

**Methods:**

This is an analytical cross-sectional study. The main outcomes were demographic and clinical characteristics of COVID 19 patients who died from March till December 2020, during the hospital stay. We collected 193 records of COVID-19 patients, from two major hospitals in Al Madinah region, Saudi Arabia. Descriptive and inferential analysis were performed to identify and relate the factors of early death.

**Results:**

Out of the total deaths, 110 died during the first 14 days of admission (Early death group) and 83 died after 14 days of admission (Late death group). Early death group had a significantly higher percentages of old age patients (p=0.027) and males (72.7%). Comorbidities were found in 166 (86%) of cases. Multimorbidity were significantly higher in early deaths than in late deaths 74.5% (p=<0.001). Women had significantly higher mean values of CHA2SD2 comorbidity scores (3.28 versus 1.89 for men; p <0.001). Moreover, predictors of high comorbidity scores were older age (p=0.005), higher respiratory rate (p=0.035), and raised alanine transaminase (p=0.047).

**Conclusion:**

Old age, comorbid illness, and severe respiratory involvement were prevalent among COVID-19 deaths. Comorbidity scores were significantly higher in women. Comorbidity was found to be significantly more associated with early deaths.

## INTRODUCTION

Coronavirus disease 2019 is a viral respiratory illness caused by SARS-CoV-2 virus.^1^ COVID-19 ranks among the leading causes of death globally.^2^ According to the world health organization (WHO) report, there were more than 248 million confirmed cases of COVID-19 including nearly five million deaths worldwide.^3^ The 22 countries of the Eastern Mediterranean Region (EMR) region, by the end of 2021, recorded more than 16 million cases which constitute about 6.7% of the global count at that time with nearly 300 thousand deaths (Case Fatality Rate CFR 1.8 %).^4^ In Saudi Arabia, there were nearly 549 thousand confirmed cases including 8800 deaths (1.6% CFR)^5^ with recorded 25814 cases (4.7% of Saudi Arabia cases) and 305 deaths in Al-Madinah Al-Monwarah city (1.17% CFR).^6^ The initially reported death rate in Saudi Arabia was 3.45% on March 24, 2020, and the highest reported was 10.8%.^7^ According to an Italian study, 53.4% died in the hospital as an unusual crisis.^8^ The demographic factors including old age and male sex, clinical, virologic, hematological, biochemical, and radiographic factors might correlate with COVID-19 disease severity.^9^

Poor outcomes in COVID-19 were linked to severe respiratory involvement, cardiac, and renal involvement.^10^ Previous reports showed that comorbidities like COPD, and diabetes mellitus were linked to poor outcomes.^11^ Significant independent potential predictors of in-hospital mortality were reported in elderly COVID-19 patients including hypernatremia [HR 9.1], lymphopenia [HR7.4], and cardiovascular disease (CVD) other than hypertension [HR6.4].^12^ Early evidence showed that an elevated neutrophil/lymphocyte ratio (NLR) [HR 2.5] was a significant predictor of severity of illness in COVID 19 along with age.^13^ However, some contradictory evidence was also found, particularly in our local population.^9^ Identification of those at risk of mortality is crucial, to strengthen preventive strategies and future planning of healthcare requirements.^14^

This study aimed to describe the demographic, clinical, and laboratory characteristics of in-hospital COVID-19 deaths in Almadinah Almonawarah city, Saudi Arabia, and to identify risk factors associated with early mortality among them. The study findings would be useful in preparing the health systems to prioritize and locate the resources according to their imminent need, towards holistic care.

## METHODS

This is a records-based analytical cross-sectional study involving mortality cases from two large hospitals (Madinah General Hospital and Ohud Hospitals), selected purposely in Al-Madinah Almonawarah city, Saudi Arabia. We included all COVID-19 PCR positive cases who died in hospital from March to December 2020.

Data of 193 deceased patients were extracted from medical records using a standard data collection form designed by the authors. We calculated CHADS2 index (congestive heart failure, hypertension, age, diabetes mellitus, stroke, or transient ischemic attacks)^15^ and CHA2DS2-VASc index (congestive heart failure, hypertension, age, diabetes mellitus, stroke, or transient ischemic attacks, sex, vascular disease)^16^ to study the association of comorbidity with early deaths. CHA2DS2-VASc risk of morbidity and mortality categories are no risk (0), low risk (1), intermediate risk (2), and high risk (≥3).^17^ Data were recorded on an Excel sheet, checked, and analyzed on SPSS version 22. Descriptive analysis was done for all qualitative and quantitative variables and inferential was done by using Chi-square test, Independent T test respectively, to assess the association of factors between early and late deaths. Logistic Regression analysis performed by applying Omnibus test model for associated factors of early deaths. Imputation analysis was carried out for the missing values.

The study was approved by the Institutional Review Board (IRB) General Directorate of Health Affairs in Madinah (Ref: #H-03-M-084). All data were anonymous and informed consent was not needed and waived as data were retrieved retrospectively.

## RESULTS

A total of 193 COVID-19 deaths were reported during hospitalization in the study period (March-December 2020). The median interval from hospitalization to death was 12 days with a range of 0-80 days. Out of them, 110 died during the first 14 days of admission (Early death group) and 83 died after 14 days of admission (Late death group). Nealy half of COVID-19 deaths were of old age [≥65 years] 98 (50.7%) with a significantly higher prevalence of old age patients in the early death group (p=0.027). Male gender was predominant in overall 134 (69.4%), in early death group 80 (72.7%), and in late death group 54 (65%) (Table-I). However, the apparent male predominance was not statistically significant.

**Table-I:** Sociodemographic, clinical and treatment profile of COVID-19 deaths.

Variable	Overall cases n=193	Early death group n=110	Late death group n=83	Significance
Age (years)	63.5±15.3	62.9±15.8	64.5±14.7	0.46
Old age ≥65 years	98(50.7)	64(58.1)	34 (45.7)	0.027*
Gender (male)	134 (69.4)	80 (72.7)	54 (65)	0.30
Saudi national	104 (57.4)	56 (50.9)	36 (43.4)	0.56
Non Saudi [ n=181]	77 (42.5 )	46(41.8)	27 (32.5)	
Any Comorbidity	166 (86)	99 (90)	67 (80.7)	0.06
Combined multimorbidity	117 (60.6)	82 (74.5)	35(42.1)	<0.001*
Diabetes Miletus	126 (65.2)	77 (70)	49 (59)	0.11
Hypertension	114 (59.1)	69 (62.7)	45 (54.2)	0.23
Coronary artery disease	23 (11.9)	11(10)	13(15.6)	0.27
Chronic kidney disease	19 (9.5)	15 (13.6)	4 (4.8)	0.042*
Neurologic disease	14 (7.1)	9(8.1)	5(6)	0.56
COPD/Asthma	19(9.8)	16 (14.5)	3 (3.9)	0.012*
CHADS2 Index	1.0 [IQR=1-2]	1.4±0.7	1.5±0.9	0.11
CHA2DS2 Index	2.0 [IQR=1-3]	2.17±1.3	2.51±1.5	0.12
Fever	122 (61)	75 (68.1)	47 (56.6)	0.09
Dyspnea	144 (74.6)	89 (89.9)	55 (66.2)	0.021*
Cough	129 (53.3)	77 (70)	52 (62)	0.28
Chest pain	18 (9.3)	9 (8.1)	9 (10.8)	0.8
Headache	13 (6.7)	10 (9.2)	3 (3.6)	0.13
Malaise/myalgia/fatigue	14 (7.2)	10 (9.2)	4 (4.8)	0.27
GI symptoms	35 (18.1)	14 (12.7)	21(25.3)	0.025*
Anticoagulants	118 (61.1)	96 (87.3)	22 (26.5)	0.000*
Steroids	117 (60.6)	58 (52.7)	59 (71.1)	0.01*
Hydroxychloroquine	71(36.7)	32 (29)	39 (46.9)	0.011*
Remdesivir	4 (2.1)	4(3.6)	00	0.07
Lopinavir/ flavipiravir	139(72)	87(79.1)	52(62.6)	0.012*

Data are presented as number (%), Mean±SD, Median [IQR]

* Significant p value, CHADS2: congestive heart failure, hypertension, age, diabetes mellitus, stroke, or transient ischemic attacks; CHA2DS2-VASc congestive heart failure, hypertension, age, diabetes mellitus, stroke, or transient ischemic attacks, sex, vascular disease; GI: gastrointestinal, IQR: interquartile range.

Comorbidities were prevalent in the reported cases, 166 (86%). Diabetes and hypertension were the most common comorbidities; Multimorbidity was significantly higher in the early death group than in the late death group (82 (74.5%) Vs. 35(42.1%); p=<0.001). Whereas, chronic kidney disease was significantly higher in the early death group (p=0.04) (Table-I). The male/female ratio with comorbidities was 2.2:1 compared to 2.3:1 without comorbidities.

**Fig.1:**
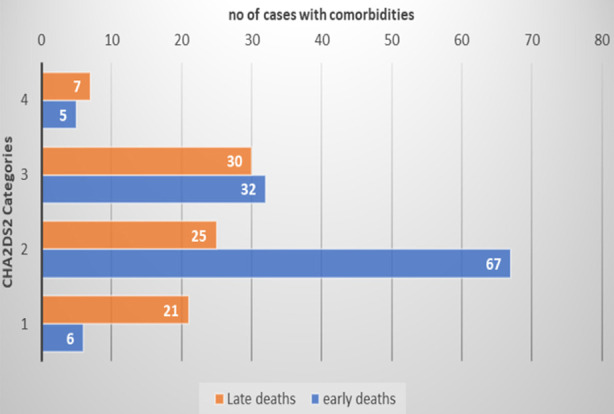
CHA2DS2 comorbidity score in early and late death group. **1=**CHA2DS2 class 0-1; **2=** CHA2DS2 class 2-4; **3=** CHA2DS2 class 4-6; **4=** CHA2DS2 class > 6, **CHA2DS2**: congestive heart failure, hypertension, age, diabetes mellitus, stroke, or transient ischemic attacks.

No significant difference in mean CHADS2 or CHA2DS2-VASc comorbidity indices between early and late death groups. However, the early death group had a higher percentage of low and intermediate risk (Fig.1). Women had significantly higher mean values of CHA2SD2-VASc comorbidity scores (3.28) compared to men 1.89 (p= < 0.001).

Moreover, predictors of high multimorbidity scores were older age (64.82 years versus 55.96 years for those without comorbidities, p=0.005), higher respiratory rate (26.04 cycle/min versus 22.75 cycle/min for those without comorbidities, p=0.035), raised alanine transaminase (ALT) (166.98 versus 76.13 for those without comorbidities, p=0.047). Besides, 60 (31%) had evidence of ARDS.

Dyspnea, fever, and cough were the most common symptoms. Dyspnea was significantly more prevalent in the early death group 89 (80.9%) versus 55 (66.2%) in the late death group (p=0.021); respiratory failure was reported in 60.6% of deaths; while gastrointestinal symptoms were significantly more prevalent in the late death group 21(25.3%) compared to the early death group 14 (12.7%) (p=0.025 (Table-I). Most patients 135 (70%) had bilateral pulmonary infiltrates in chest radiographs, and it was not significantly different in the two groups.

Abnormal laboratory parameters were present in most cases are shown in Table-II. Abnormal high serum creatinine values of more than 106 mmol/l were found in 102 (57.6%) of the cases, and abnormal high bilirubin values (>1.2 mg/dl were detected in three-quarters of cases 143 (74.1%). High ALT and AST were seen in 74 (44%) and 54 (31.5%) respectively. Leucocytosis was detected in 108 (62%) of the cases while neutropenia and lymphopenia were also prevalent (36 (66.6%), 34 (30.6%) respectively. Women had a significantly lower mean value of hemoglobin (10.69 gm/dl versus 12.07 gm/dl for men; p=0.04), while men had significantly higher mean values of creatinine (228.92 mmol/L versus 151.64 mmol/L for women, p=0.033). Analysis of variances using the Omnibus test of model correlation showed significant correlation between length of survival and both comorbidity (OR 2.92) and NLR (OR 1.97) (Table-III).

**Table-II:** Baseline Vital signs & biochemical findings of cases.

Variable	Overall n=193	Early death group n=110	Late death group n=83	Sig.
Temperature (°C)	37±.7	37±.70	37.20±.60	0.070
Heart rate (median) (beat/min)	94±20	94±24	93±18	0.920
Respiratory rate (breaths/min)	25±7	26.7±7	23.9±6	0.008*
Mean Arterial Pressure (mmHg)	92.5±17	89.6±17	96.4±16	0.007*
SpO2 %	87.9±12.5	87.9±13	88±11	0.960
PaO2 (mmHg)	50.4±29.3	52.4±19	48.7±19	0.200
PaO2/FiO2	396.7±158.3	424±164	356±140	0.009*
PaCO2 (mmHg)	44.1± 13.8	42.7±13	45.8±14	0.120
pH	7.30±0.35	7.3±.4	7.3±.1	0.880
Hemoglobin (g/dl)	11.6±2.8	12.1±2.7	10.9±2.8	0.008*
HCO3 (mmol/L)	22.6±6.40	21.8±6.1	23.7±6.6	0.046*
Platelets (x10^3^)	215±126	218±127	210±125	0.680
WBCs (x10^3^)	12.6±7.3	12.5±7.9	12.6±6	0.900
NLR	20±30 12. [6.2-26.2]	19±36 14.5 [6.9-30.9]	22±20 16.5[11-37.7]	^δ^0.410 0.05*
INR	1.4±1.0	1.4±1.2	1.43±.5	0.86
ALT (IU/L)	48.5 [31.2- 84.2]	43 [24.5-76.5]	53.5[33.5-102]	0.35
AST (IU/L)	55 [36.2- 87.7]50	64[41.5-86]	54.5[28.5-90.2]	0.82
Bilirubin (mmol/L)	12.8 [8.0- 21.3]	12.3 [7.6-16.4]	13.6[10-20.9]	0.37
Prothrombin time (sec)	14 [12.8-15.9]	13.9[12.5-15.5]	14.7[12.9-16.8]	0.98
BUN (mmol/L)	12.4 [6.5- 22.8]	9.8[5.7-21.4]	15.6[7.9-29]	0.06
Creatinine (mmol/L)	117.3 [76.2-225]	119[75.4-271.5]	95.5[76.2-239]	0.93
Glucose (mmol/L)	10.3 [6.9- 13.6]	9.6 [6.1-14.3]	12.1[7.4-13.5]	0.12
LDH (IU/L)	555[501-725]	659[450-850]	522[389-726]	0.21

Data are presented as mean±SD or median [IQR], δIndependent sample median test applied,

* Significant p-value FIO2: fraction of inspired oxygen; PaO2: partial pressure of oxygen; PaCO2: partial pressure of carbon dioxide; NLR:neutrophil Lymphocyte ratio ; INR:international normalization ratio; LDH: lactate dehydrogenase; ALT: alanine transaminase; AST: aspartate transferase; RBC: red blood cells; WBC: white blood cells.

**Table-III:** Logistic Regression analysis .

Variable	B	SE	Wald	Sig.*	OR
Comorbidity	1.058	0.493	4.840	0.028	2.961
CHA2DS2	-0.120	0.118	1.029	0.310	0.887
PaO2/FiO2	0.001	0.001	3.178	0.075	1.001
NLR	-0.230	0.009	6.390	0.011	1.978
Hg	0.030	0.051	0.356	0.551	1.031

CHA2DS2: congestive heart failure, Hypertension, age, Diabetes mellitus, Stroke or transient ischemic Attacks, Sex, Vascular Disease; NLR: neutrophils/lymphocytes ratio, Hg: hemoglobin, PaO2/FiO_2_: Ratio of fraction of partial pressure and of inspired Oxygen.

* Omnibus test of model coefficient.

## DISCUSSION

The main finding of this study is the prevalence of old age 98(50.7%) among COVID-19 deaths with a significantly higher prevalence of old age in the early death group (p=0.027). Previous studies reported age more than 65 to be a strong predictor of mortality.^17^-^19^ High mortality in the middle-aged and elderly may be attributed to weak physical resistance and more susceptibility to comorbid illness.^20^ Below 30 years group contribute a small percentage to our study, this may be explained by the fact that during the early phases of COVID-19 childhood deaths were rare. This may be attributed to low lung expression of ACE2 receptors in children.^21^ Male gender was prevalent in overall deaths (69.4%) with no significant difference between early deaths and late deaths as shown in other studies as well.^20^,^22^ This may be due to the effect of sex hormones^23^ and more expression of angiotensin receptor-2 (ACE2) in males.^24^ Estrogen may have a protective effect in women.^25^,^26^

We found a high percentage of comorbidities (86%) and multimorbidity were significantly higher in early deaths than in late deaths (74.5%) Vs. (42.1%). The early death group had a significantly higher percentage of cases with CHA2DS2-VASc scores low and intermediate risks while the late death group has a higher percentage of no risk category. Previous studies^27^,^28^ have reported that high comorbidity scores were associated with increased risk of severe COVID-19, poor outcomes, and increased mortality. Some reports found that CHA2DS2-VASc at admission is a useful tool^29^ and is a strong predictor of in-hospital mortality. Moreover, predictors of high comorbidity score were older age, higher respiratory rate, raised ALT. Previous reports showed that comorbidities were linked to more severe disease and poor outcomes^30^ and cardiovascular comorbid illness was linked to high mortality.^18^ Hypertension was associated with more severe disease and high mortality.^28^

Among comorbidities, chronic kidney disease (CKD) contributed to 9.5% of cases in this study with a significantly higher percentage in early deaths as previously reported CKD was a major predictor of mortality^28^ and acute kidney injury is common in COVID-19 patients^31^ among 50% of those admitted to ICU.^32^ The relation between CKD and COVID-19 infection may be attributed to the abnormal immune response in patients with CKD.^33^–^35^ It was evident that 9.8% of deaths in this study had COPD/Asthma (with a higher percentage in early deaths (p=0.012) (Table-I), COPD was reported as a risk factor for severe disease, ICU admission, and invasive mechanical ventilation in COVID-19.^28^

In this study respiratory involvement was more prevalent in the early death group while gastrointestinal involvement was more prevalent in the late-death group (Table-I, II). Severe respiratory involvement identified as respiratory failure 60.6%, low PaO2/FIO2 ratio, and bilateral radiographic pulmonary infiltrates were seen at the time of admission. Similar results were reported from Wuhan where 61% of ICU patients had respiratory failure at admission.^26^ This finding was linked to more severe diseases, rapid progression, and increased mortality.^28^ Previous reports found that acute respiratory distress syndrome (ARDS) develops in more than three-quarters of COVID cases requiring ICU and in more than one-third of COVID pneumonia.^36^ This new finding highlights the importance of proper and timely intervention in COVID-19 patients with severe respiratory affection. Besides gastrointestinal symptoms at presentation can be an index of suspicion for early testing and management.

The results of the current study and previous reports indicate that the total CHA2DS2-VASc score rather than individual comorbidity is a predictor of morbidity and mortality. So, CHA2DS2-VASc could be used as a prognostic factor for risk assessment at hospital admission to detect those at high risk and initiate rapid management and close follow-up.

Abnormal laboratory parameters were present in most cases in our study. We found a high mean creatinine level and elevated creatinine at baseline in 102 (57.6%) of cases, these findings support previous reports describing acute kidney injury in COVID-19 cases.^37^,^38^ This may be attributed to the effect of cytokine storm and the predominance of ACE2 receptors in the kidneys.^39^-^42^ Besides, our cases showed evidence of liver injury [high serum bilirubin (74.1%) and elevated liver enzymes (40%). Similar results had been reported by other researchers.^43^ Liver toxicity may be due to direct viral toxicity probably linked to overexpression of ACE2 receptors in the liver or viral-induced T-cell cytotoxicity.^44^ Leukocytosis and thrombocytopenia were present in 62% and 28.3% of cases respectively. Thrombocytopenia indicates severe inflammatory response and activation of the coagulation cascade with risk of thromboembolic events.^45^ Another study reported a significantly low serum albumin in expired patients with COVID-19 with strong negative correlation. Serum albumin is one of the indicators of inflammatory response^46^ and liver injury

The findings of this study suggest that older age, comorbidities, and respiratory involvement are common findings in the early death group. Older age and neutrophil lymphocyte ratio are potential predictors for early mortality. This can be utilized for triage and prioritization in hospital or ICU admission. Comorbidity scores are useful risk assessment tools for COVID-19 patients

### Limitations:

This study has some limitations, deaths out of hospital were not included and survival analysis could not be performed due to lack of control group.

## CONCLUSION

Old age, and comorbid illnesses are more common among early COVID-19 deaths. Comorbidities and neutrophils/lymphocytes ratio were strongly associated with early mortality.

It is recommended that clinicians and healthcare workers need to identify COVID-19 patients at risk of severe disease and death for timely and proper intervention. Risk assessment scores can be introduced for the triage of COVID-19 cases as prognostic indicator of morbidity and mortality. Public health authorities should exert more efforts to increase awareness of the elderly and those with comorbidities to seek medical advice early and to follow infection prevention measures to avoid catching COVID-19 disease. Future studies are required to investigate predictors of morbidity and mortality including in and out of hospital deaths.
